# Plasma membrane H^+^‐ATPase activation increases global transcript levels and promotes the shoot growth of light‐grown Arabidopsis seedlings

**DOI:** 10.1111/tpj.70034

**Published:** 2025-02-07

**Authors:** Satoru Naganawa Kinoshita, Kyomi Taki, Fumika Okamoto, Mika Nomoto, Koji Takahashi, Yuki Hayashi, Junko Ohkanda, Yasuomi Tada, Iris Finkemeier, Toshinori Kinoshita

**Affiliations:** ^1^ Institute of Plant Biology and Biotechnology University of Muenster Muenster Germany; ^2^ Graduate School of Science Nagoya University Nagoya Japan; ^3^ Center for Gene Research Nagoya University Nagoya Japan; ^4^ Institute of Transformative Bio‐Molecules (ITbM) Nagoya University Nagoya Japan; ^5^ Institute of Agriculture Shinshu University Nagano Japan

**Keywords:** acid growth, Arabidopsis, PM H+‐ATPase, shoot growth, thaliana, transcriptomics

## Abstract

Plant cell growth requires the elongation of cells mediated by cell wall remodelling and turgor pressure changes. The plasma membrane (PM) H^+^‐ATPase facilitates both cell wall loosening and turgor pressure changes by acidifying the apoplast of cells, referred to as acid growth. The acid growth theory is mostly established on the auxin‐induced activation of PM H^+^‐ATPase in non‐photosynthetic tissues. However, how PM H^+^‐ATPase affects the growth in photosynthetic tissues of Arabidopsis remains unclear. Here, a combination of transcriptomics and cis‐regulatory element analysis was conducted to identify the impact of PM H^+^‐ATPase on global transcript levels and the molecular mechanism downstream of the PM H^+^‐ATPase. The PM H^+^‐ATPase activation increased transcript levels globally, especially cell wall modification‐related genes. The transcript level changes were in PM H^+^‐ATPase‐dependent manner. Involvement of Ca^2+^ was suggested as CAMTA motif was enriched in the promoter of PM H^+^‐ATPase‐induced genes and cytosolic Ca^2+^ elevated upon PM H^+^‐ATPase activation. PM H^+^‐ATPase activation in photosynthetic tissues promotes the expression of cell wall modification enzymes and shoot growth, adding a novel perspective of photosynthesis‐dependent PM H^+^‐ATPase activation in photosynthetic tissues to the acid growth theory that has primarily based on findings from non‐photosynthetic tissues.

## Significance Statement

Plasma membrane (PM) H^+^‐ATPase activation promotes the expression of cell wall‐related genes and the growth in light‐grown Arabidopsis seedlings. This finding may provide the novel physiological role of photosynthesis‐driven activation of PM H^+^‐ATPase in photosynthetic tissues, a recently emerged regulation type of PM H^+^‐ATPase.

## INTRODUCTION

Plant growth is mediated by the expansion and division of individual cells. Expansion and division processes are linked to the structure and property of the cell wall, a rigid yet flexible barrier surrounding plant cells. The dynamic equilibrium between rigidity and flexibility is crucial for plant development (Cooper, 2000). The remodelling and loosening of the wall structures maintain flexibility. For instance, the remodelling involves the enzymes that change the properties of cellulose tethering such as the xyloglucan and pectin network (Miedes et al., 2013). The loosening of the cell wall is mediated by a decrease in apoplastic pH, activating a cascade of enzymatic reactions that loosen the cell wall structure (Cosgrove, 2024). The apoplastic acidification model is also called the acid growth model. The acid growth model has been based on extensive studies from 1970s (Hager et al., 1971; Rayle & Cleland, 1970) and the model illustrates that the phytohormone auxin induces the apoplastic acidification by activating plasma membrane (PM) P‐type H^+^‐ATPase, a primary source of H^+^ gradient across PM (Hager, 2003; Palmgren, 2001; Takahashi et al., 2012). An application of a unique fungal phytotoxin Fusicoccin, an irreversible activator of PM H^+^‐ATPase, has revealed that sole activation of PM H^+^‐ATPase can achieve the growth of cells, further illustrating the central role of PM H^+^‐ATPase in the acid growth model (Kutschera & Schopfer, 1985). Other hormones also control the apoplast acidification and cell growth, that is, brassionsteroids (BRs) promote PM H^+^‐ATPase activity (Minami et al., 2019).

Phytohormone‐mediated PM H^+^‐ATPase activity regulation consists of two distinct mechanisms. Upon the perception of the phytohormone, both types of regulation are achieved by post‐translational modification of PM H^+^‐ATPase autoinhibitory domains (Miao et al., 2022). Both the N‐terminal and C‐terminal domains of the PM H^+^‐ATPase regulate the PM H^+^‐ATPase activities (Ekberg et al., 2010; Lin *et al*., 2024). The C‐terminal autoinhibitory domain has several phosphorylation sites that control the activity of the PM H^+^‐ATPase (Falhof et al., 2016; Fuji et al., 2024; Hayashi et al., 2024). Taking auxin as one example, perception of auxin at the PM is mediated by auxin binding protein (ABP) and PM‐localised transmembrane kinases (TMKs), phosphorylating PM H^+^‐ATPase (Friml et al., 2022; Li, Verstraeten, et al., 2021; Lin et al., 2021). On the contrary, the perception of the auxin in the nucleus is mediated by the TIR1/AFB complex, which induces the expression of *small auxin‐up RNAs* (*SAURs*) family genes (Ang & Østergaard, 2023). SAURs proteins interact with protein phosphatase 2C D‐clade (PP2C‐D) family proteins and inhibit the phosphatase activity of PP2C‐D, maintaining the phosphorylation status of PM H^+^‐ATPase (Ren & Gray, 2015).

Other than phytohormonal regulation, recent studies in photosynthetic tissues have discovered that photosynthesis promotes the activation of the PM H^+^‐ATPase via phosphorylation of C‐terminus residues (Hayashi et al., 2024; Kinoshita et al., 2023; Okumura et al., 2016). The activation of the PM H^+^‐ATPase in leaves is dependent on the photosynthetic activity and the photosynthesis product, but independent from the light signalling mediated by known photoreceptors, that is, phytochrome, phototropin, nor cryptochrome (Okumura et al., 2016). Further investigation on the activation mechanism of photosynthesis‐dependent PM H^+^‐ATPase has found a novel activator of PM H^+^‐ATPase in light‐illuminated and sugar‐fed leaves, SAUR30 (Kinoshita et al., 2023), that is not responsive to external auxin application (Paponov et al., 2008). The regulatory mechanism of PM H^+^‐ATPase by photosynthesis raises a fundamental question: What physiological roles does photosynthesis‐dependent PM H^+^‐ATPase activation play in photosynthetic tissues? Nitrate uptake into leaves has been proposed as one role of the photosynthesis‐dependent activation of PM H^+^‐ATPase (Kinoshita et al., 2023). In addition, it is noteworthy that an acid growth model has been established in divergent plant species, primarily using coleoptile, hypocotyl of etiolated seedlings or roots, non‐photosynthetic tissues. However, the growth of cells in photosynthetic tissues such as leaves and light‐grown seedlings, green seedlings, has a limited investigation; in other words, how PM H^+^‐ATPase activity promotes growth in photosynthetic tissues remains elusive. In addition, while transcriptional inhibition is suggested to suppress the acid growth of cells (Arsuffi & Braybrook, 2018), the impact of PM H^+^‐ATPase activation alone—excluding light or phytohormone—on global transcript levels in photosynthetic tissues remains unknown.

Here, we investigated the impact of PM H^+^‐ATPase activation on the growth of green seedlings and the global transcriptome, finding that PM H^+^‐ATPase activation promotes the growth of green seedlings via upregulation of cell wall‐related gene expression. Further *in silico* analysis using the promoters of PM H^+^‐ATPase activation‐responsive genes predicted the involvement of Ca^2+^ signalling, which was confirmed with observation using the Ca^2+^ biosensor, GCaMP3 (Toyota et al., 2018). From the results, we propose a novel perspective in the acid growth model, implying that PM H^+^‐ATPase activation in photosynthetic tissues promotes the growth of cells by indirectly inducing cell wall‐related gene expressions. Considering the PM H^+^‐ATPase is activated mainly by photosynthesis and photosynthetic products, our findings in photosynthesis tissues suggest one of the physiological roles of photosynthesis‐dependent activation of PM H^+^‐ATPase, shedding light on a novel viewpoint of photosynthesis in the framework of the acid growth theory.

## RESULTS

### PM H^+^‐ATPase promoted the growth of green seedlings

**Figure 1:**
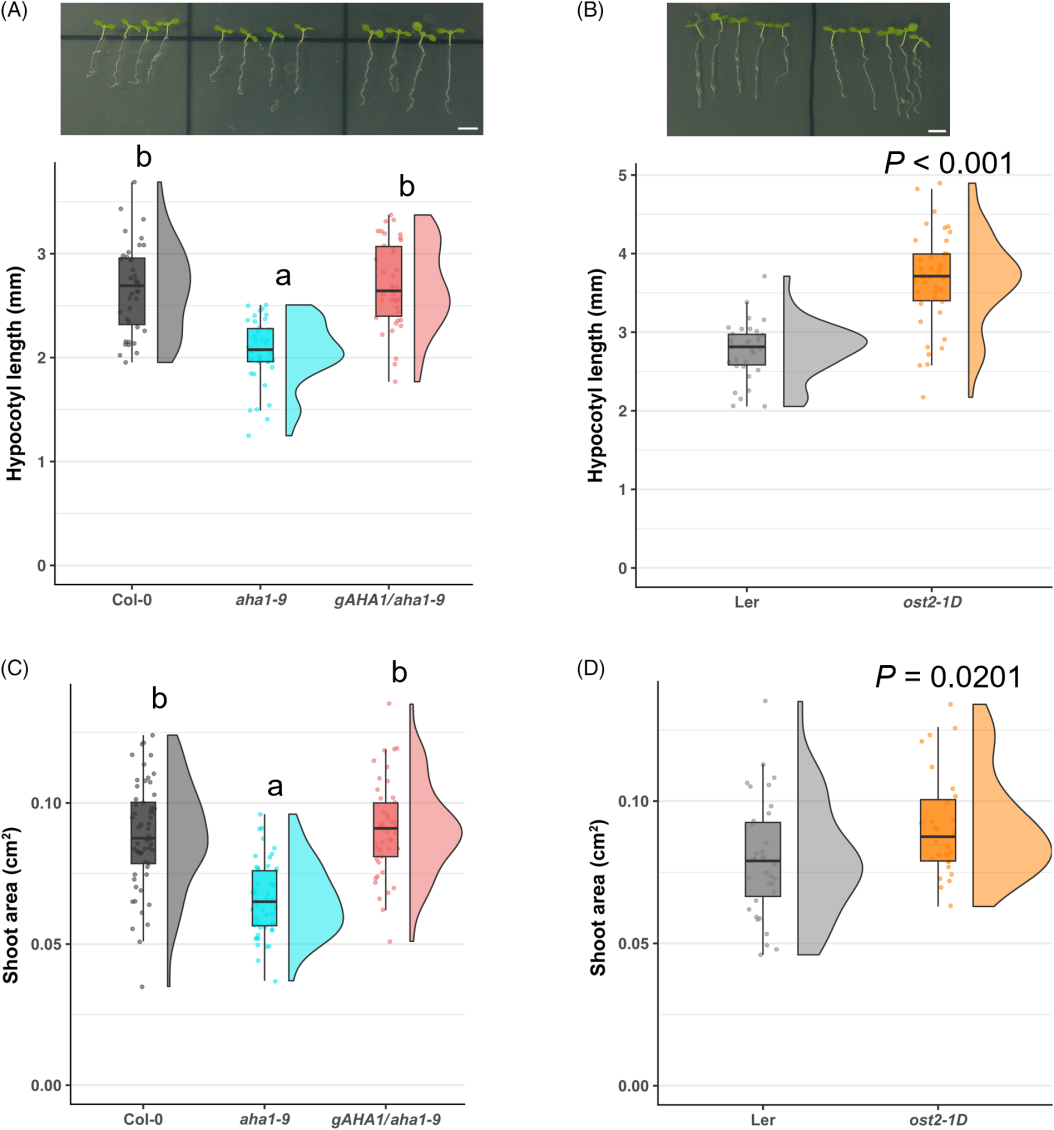
Hypocotyl elongation of seedlings with different PM H^+^‐ATPase activity. (A) Hypocotyl length and (C) shoot area (cotyledons, main leaves, and a part of hypocotyl) of wild‐type (Columbia‐0, Col‐0), *aha1‐9*, complementation line *gAHA1*/*aha1‐9*. (B) Hypocotyl length and (D) shoot area of wild‐type (Landsberg erectra, Ler) and *aha1* mutant with constitutive activation mutation (*open stomata 2–1 dominant*, *ost2‐1D*). (A) and (B) Each dot indicates the length of one seedling, and the boxplot and half‐violin plot present the distribution and density of hypocotyl length; Scale bars: 5 mm. (A) *n* = 38–39. Different letters above boxplot indicate the significant difference, determined by one‐way ANOVA with Tukey HSD. (B) *n* = 34–42. The *P*‐value was determined by Welch's *t*‐est. (C) and (D) Each dot indicates the shoot area of one seedling, and the boxplot and half‐violin plot present the distribution and density of the shoot area. (C) *n* = 45–56. Different letters above the boxplot indicate the significant difference, determined by one‐way ANOVA with Tukey HSD. (D) *n* = 28–35. The *P‐*value was determined by Welch's *t*‐test.

To test whether PM H^+^‐ATPase activity influences the shoot growth of green seedlings, the hypocotyl length and the shoot area in loss‐of‐function mutant and constitutive active mutant of AHA1 were measured. The shoot area of each seedling consisted of mainly the cotyledon, newly emerging leaves, and a small part of the hypocotyl. The loss‐of‐function mutant, *aha1‐9*, showed reduced growth compared to wild‐type Col‐0 and the growth was recovered by the complementation of AHA1 in *aha1‐9, gAHA1*/ *aha1‐9* (Figure 1A,C; Figure S1). On the contrary, the growth of the AHA1 constitutive active mutant, *open stomata 2–1 dominant* (*ost2‐1D*), showed a higher growth compared to wild‐type *Landsberg erecta* (Ler) (Figure 1B,D). These results illustrated that the PM H^+^‐ATPase activity plays a significant role in the shoot growth of green seedlings.

### Global transcript expression was affected by PM H^+^‐ATPase activation

**Figure 2:**
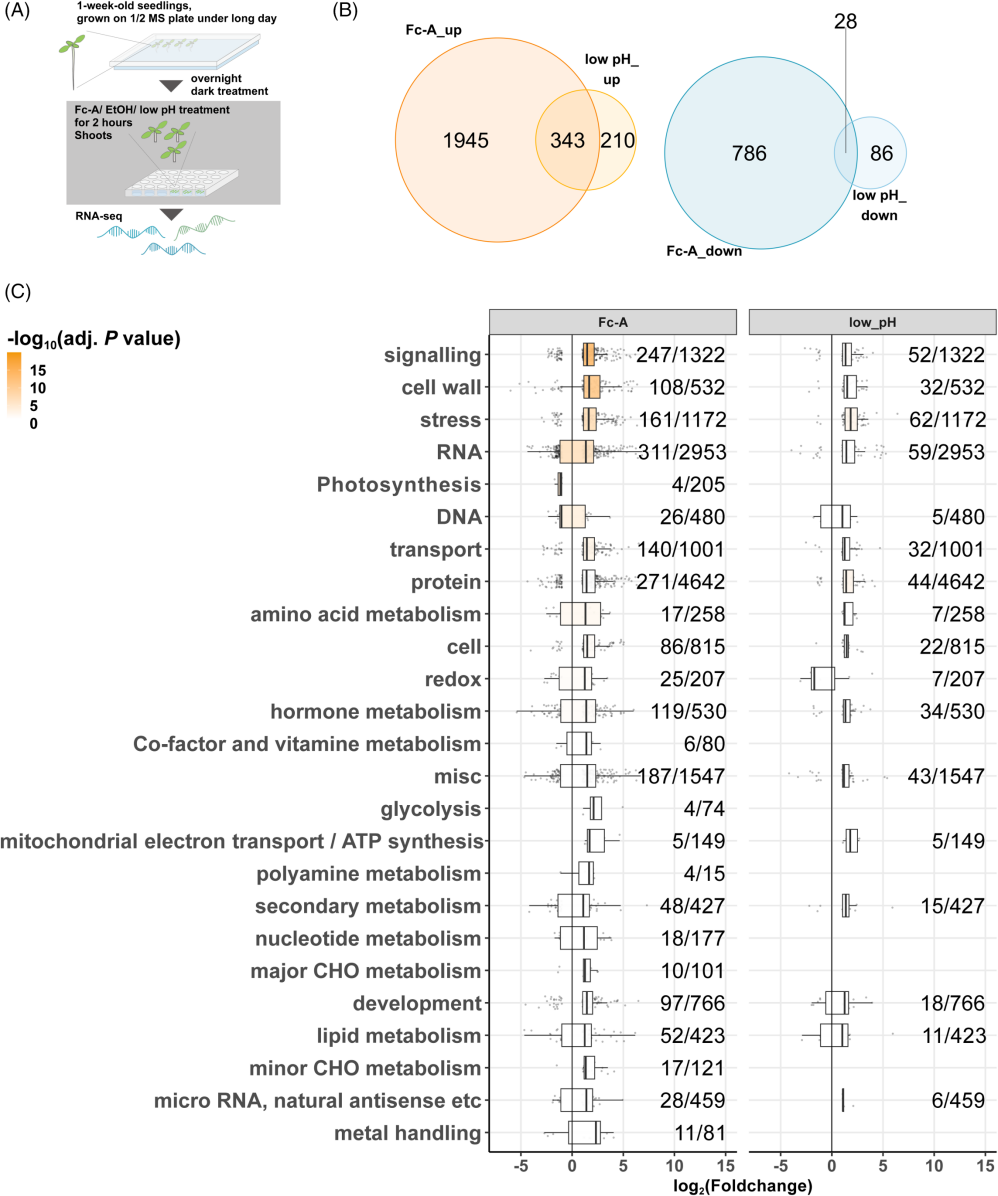
Transcriptome changes in Fc‐A‐treated seedling shoots. (A) Schematic procedure of treatment and sampling conditions. One‐week‐old seedlings were kept in the dark overnight and then incubated in liquid media with 30 μM Fc‐A or an equal amount of ethanol for 2 h. (B) Venn diagrams represent the numbers of differentially expressed genes (DEGs; |foldchange| >2, FDR = <0.05) in Fc‐A treated or low pH media‐treated seedlings compared to ethanol‐treated seedlings. On the left are upregulated DEGs, and on the right are downregulated DEGs. (C) The boxplots represent the distribution of DEGs expression changes in functional category groups compared to the ethanol‐treated samples. The numbers beside the boxplots indicate the number of DEGs over the number of all registered genes in the category groups. The Whitney *U* test in MapMan determined adjusted *P*‐values.

To investigate the mechanism of PM H^+^‐ATPase‐dependent growth in green seedlings, the changes in transcript levels were detected by RNA‐seq. Seedlings were kept in the dark overnight to reduce the activity of PM H^+^‐ATPase (Kinoshita et al., 2023; Okumura et al., 2016) and then incubated in liquid media with mock ethanol (EtOH) or a PM H^+^‐ATPase activator, Fusicoccin‐A (Fc‐A) (Figure 2A). Fc‐A causes the activation of PM H^+^‐ATPase via glueing the interaction of 14–3‐3 protein and penultimate threonine residue (Thr) of PM H^+^‐ATPase C‐terminus (Fuglsang et al., 2003; Kiriyama et al., 2024). Due to the high price of commercially available Fc‐A that is unsuitable for mass consumption, this study extracted and purified Fc‐A from a fungus, *Phomopsis amygdali* (Sassa et al., 2002). Comparing ethanol‐ and Fc‐A‐treated seedlings in the dark can elucidate the transcriptional changes downstream of PM H^+^‐ATPase activation in the seedlings. Since the activation of PM H^+^‐ATPase lowers pH in the apoplast, seedlings treated by low pH liquid media were included for the RNA‐seq analysis. Interestingly, activation of PM H^+^‐ATPase by Fc‐A induced considerable changes in transcript levels compared to the low pH treatments (Figure 2B; Data S1), implying the PM H^+^‐ATPase activation stimulated the divergent response in plant cells. Transcript levels in some gene functional groups determined by MapMan community (Thimm et al., 2004) were significantly different, especially the genes that have roles in signalling, cell wall, and stress (Figure 2C). A more specific grouping of the genes showed the genes involved in cell wall modification (Figure S2). As the gene expressions in the stress category were different, the transcriptome dataset from other biotic/abiotic stress‐treated samples was compared. The genes in the stress category as well as the signalling category showed similar trends as other stress conditions with the numbers of DEGs reaching 13%–18% of the registered genes in the categories (Figure S3; Darwish et al., 2022; Ding et al., 2022; Safaeizadeh et al., 2024). Uniquely to the Fc‐A treatment, the genes in the cell wall and the cell wall‐related categories showed uniform upregulation and distinctly higher numbers of DEGs in the category, 19.7% in the cell wall category (Figure S3) and 44.4% in the cell wall protein AGPs category (Figure S4). Moreover, the upregulation of sugar transporters and trehalose 6‐phosphate phosphatase were observed, rather uniquely in the Fc‐A treated samples (Figure S4).

Since Fc‐A also activates the potassium channel KAT1 in planta (Saponaro et al., 2017), the application of Fc‐A may induce side effects on the transcriptional changes. To minimise the possibility of picking up the genes from the side effect, previous transcriptome datasets from light‐illuminated and sucrose‐fed leaves were compared as reference datasets because light illumination and sucrose feeding induce PM H^+^‐ATPase activity in leaves, which should contain the genes both upstream and downstream of PM H^+^‐ATPase activation (Kinoshita et al., 2023). GO enrichment analysis on the transcripts that were induced by Fc‐A treatment in seedlings, light illumination and sucrose feeding on leaves showed significant enrichment in genes involved in cell wall biogenesis and glucosyl modification (Figure S5), suggesting that the PM H^+^‐ATPase activation in green tissues promotes the transcript level of cell wall‐related genes and therefore the promote the cell growth by modifying the cell wall property. It should be noted that similar GO enrichment in cell wall modification genes has been observed in the low pH‐treated root of Arabidopsis (Lager et al., 2010). However, our transcriptome analysis identified specific genes in green seedlings or novel genes involved in PM H^+^‐ATPase activation‐dependent cell wall genes (Figure S6).

### Cell wall modification‐related genes were induced in PM H^+^‐ATPase activity‐dependent manner

**Figure 3:**
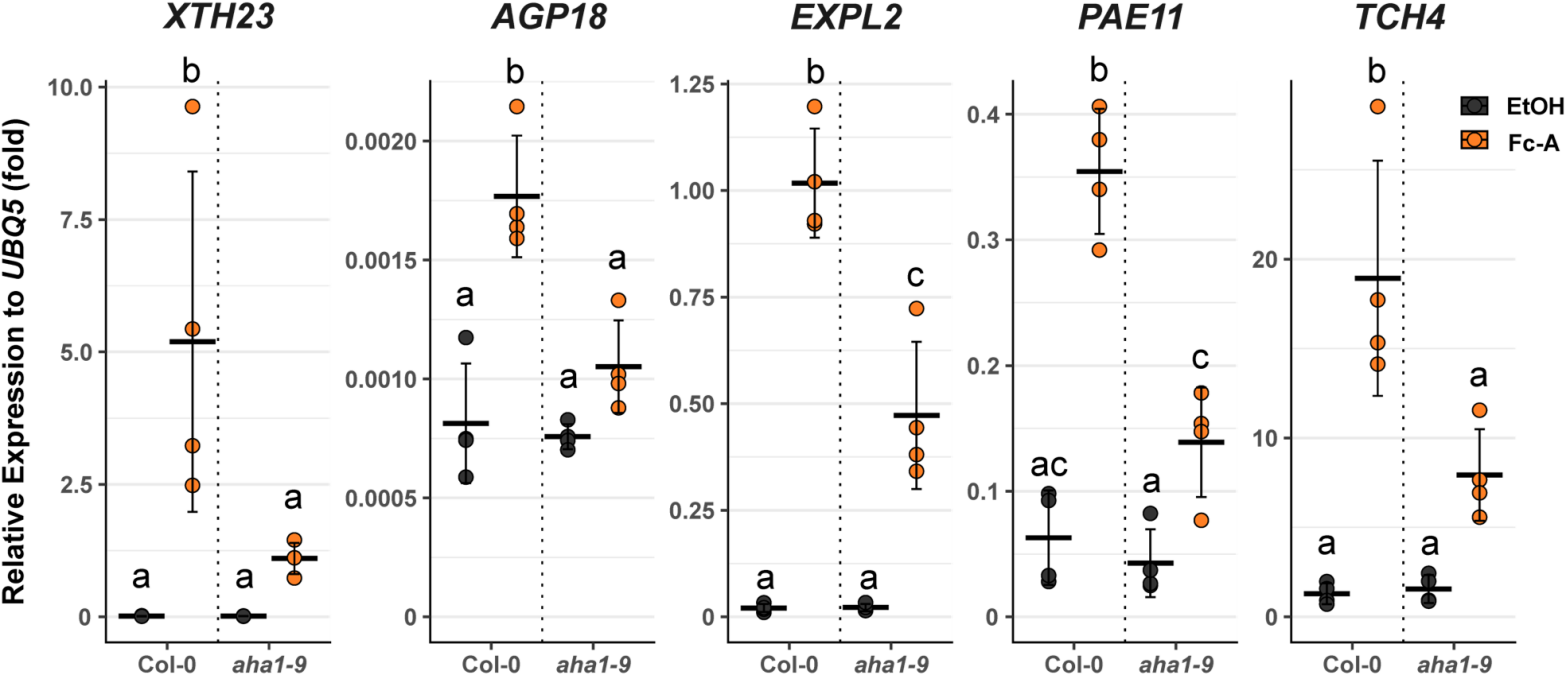
PM H^+^‐ATPase‐dependent expression changes of cell wall‐related genes. The relative expression of representative cell wall‐related genes to ubiquitous *UBQ5* in wild‐type (Col‐0) and *aha1‐9*, determined by RT‐qPCR. Each point represents one biological replicate. Cross‐bars and error bars represent the mean ± SD in each condition. Different letters above error bars indicate the significant difference in each gene expression, determined by one‐way ANOVA with Tukey HSD.

To further confirm whether the cell wall modification genes induced by Fc‐A depend on PM H^+^‐ATPase, the changes in transcript levels were checked by RT‐qPCR. As expected, the induction of all tested cell wall‐related genes was significantly reduced in the *aha1‐9* mutant upon Fc‐A treatment (Figure 3), indicating that the AHA1 plays an essential role in inducing the cell wall‐related gene expression. In addition, Fc‐A transcriptome analysis revealed that the expression level of PM H^+^‐ATPase activator, *Small Auxin Up RNA 30* (*SAUR30*), was induced by Fc‐A treatment, and the *SAUR30* gene expression induction by Fc‐A was reduced in *aha1‐9* (Figure S7A). A recent study has revealed that SAUR30 is a positive regulator in photosynthetic product‐dependent activation of PM H^+^‐ATPase (Kinoshita et al., 2023). Our results suggest that SAUR30 regulates PM H^+^‐ATPase in a positive feedback manner.

### Putative CAMTA‐binding pentamer was enriched in the promoters of PM H^+^‐ATPase‐ and low pH‐driven DEGs

**Figure 4:**
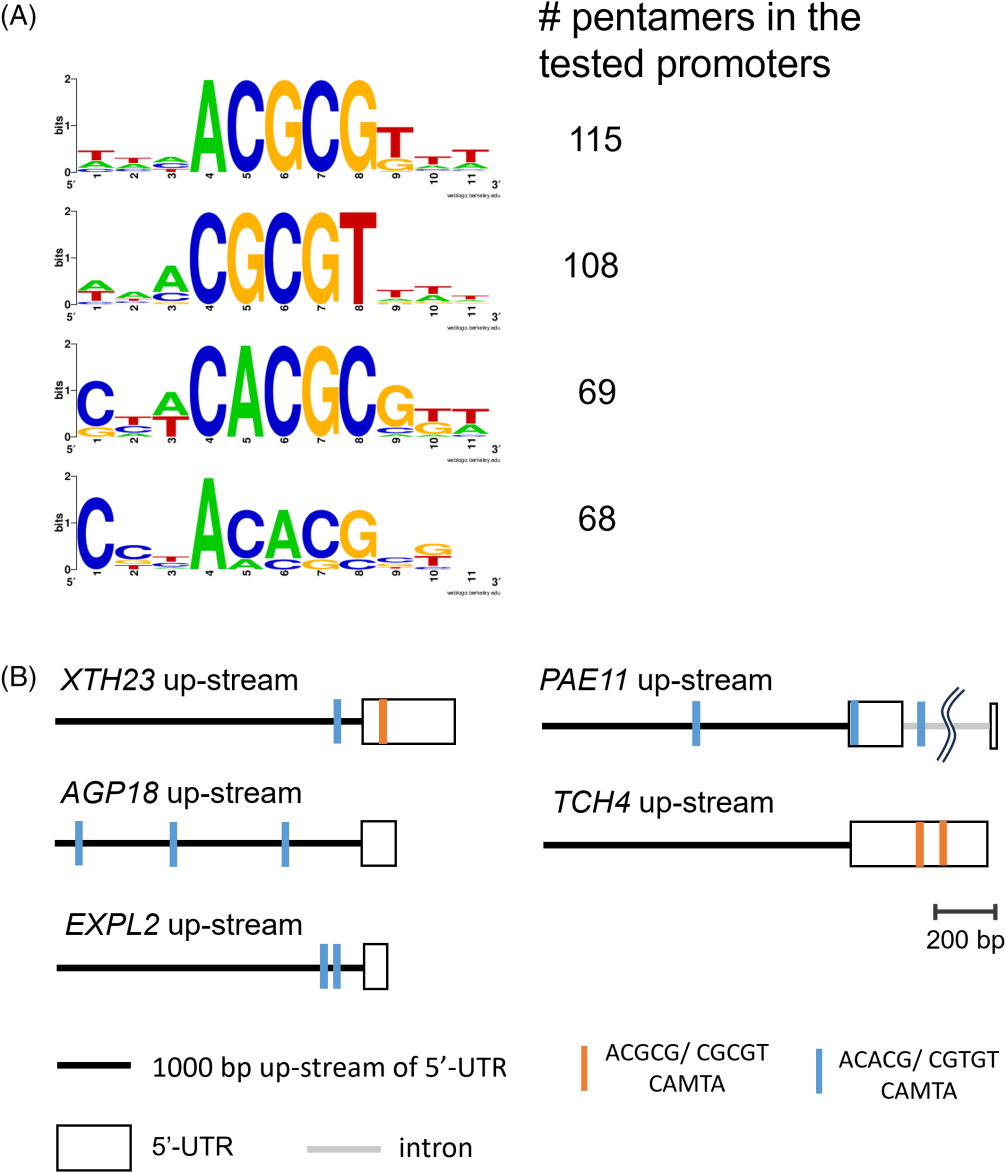
Frequently appeared pentamer in Fc‐A and low pH treatment. (A) The frequently observed pentamers were searched in the 1000 bp upstream of TOP200 most Fc‐A and low pH‐induced genes' transcription start site. Numbers next to the motif logos represent the numbers of pentamers in the datasets. The top 4 most observed pentamers, mostly CAMTA‐binding motif GCGC box [CGC(/T)GT], are listed. (B) Schematic diagram of the pentamer positions in the promoter region of cell wall‐related genes. The black line represents the 1000 bp upstream of 5′‐UTR; blue narrow line and orange narrow line indicate the position of the GCGC box, ACGCG and ACACG pentamer, respectively; the white box represents the position of 5′‐UTR.

To investigate how PM H^+^‐ATPase activation affects the global transcript levels, 1000 bp upstream of the transcription start site of the genes that were highly increased upon Fc‐A and low pH medium treatment were extracted from the database and then computationally analysed how frequently specific pentamer can be found in the datasets (Matsumura et al., 2022). The input genes were the top 200 most Fc‐A‐induced genes out of genes that were increased upon Fc‐A and low pH treatment. Interestingly, the three most frequently observed pentamers in the tested promoters were similar to the calmodulin‐binding transcription activator (CAMTA) binding cis‐elements, CACGC or CGCGT (complementary, ACGCG) (Figure 4A). Most tested cell wall‐related genes had the same pentamers in their 1000 bp upstream of 5′‐UTR, except TCH4 had the pentamer within 5′‐UTR (Figure 4B) as well as SAUR30 (Figure S7B). In addition, the comparison of our transcriptomics results with the previously detected cell wall‐related gene expression in *camta* mutants over wild‐type showed mostly reversed patterns (Figure S8; Kim et al., 2013). However, a comparison of stress‐responsive transcription factor expression change in various treated samples revealed that Fc‐A‐ nor low pH‐treated samples did not show a significant upregulation of stress‐responsive DREB1B (Figure S9; Darwish et al., 2022; Ding et al., 2022; Safaeizadeh et al., 2024). Considering that the expression of DREB1B is also regulated by CAMTA under the cold acclimation process (Kidokoro et al., 2017), those results suggest that the Fc‐A treatment involves the activation of CAMTA but with an unknown regulatory factor of CAMTA and, therefore, induces the expression of cell wall‐related genes (See *Discussion*).

### PM H^+^‐ATPase activation increased cytosolic Ca^2+^ levels in the hypocotyl of green seedlings

**Figure 5:**
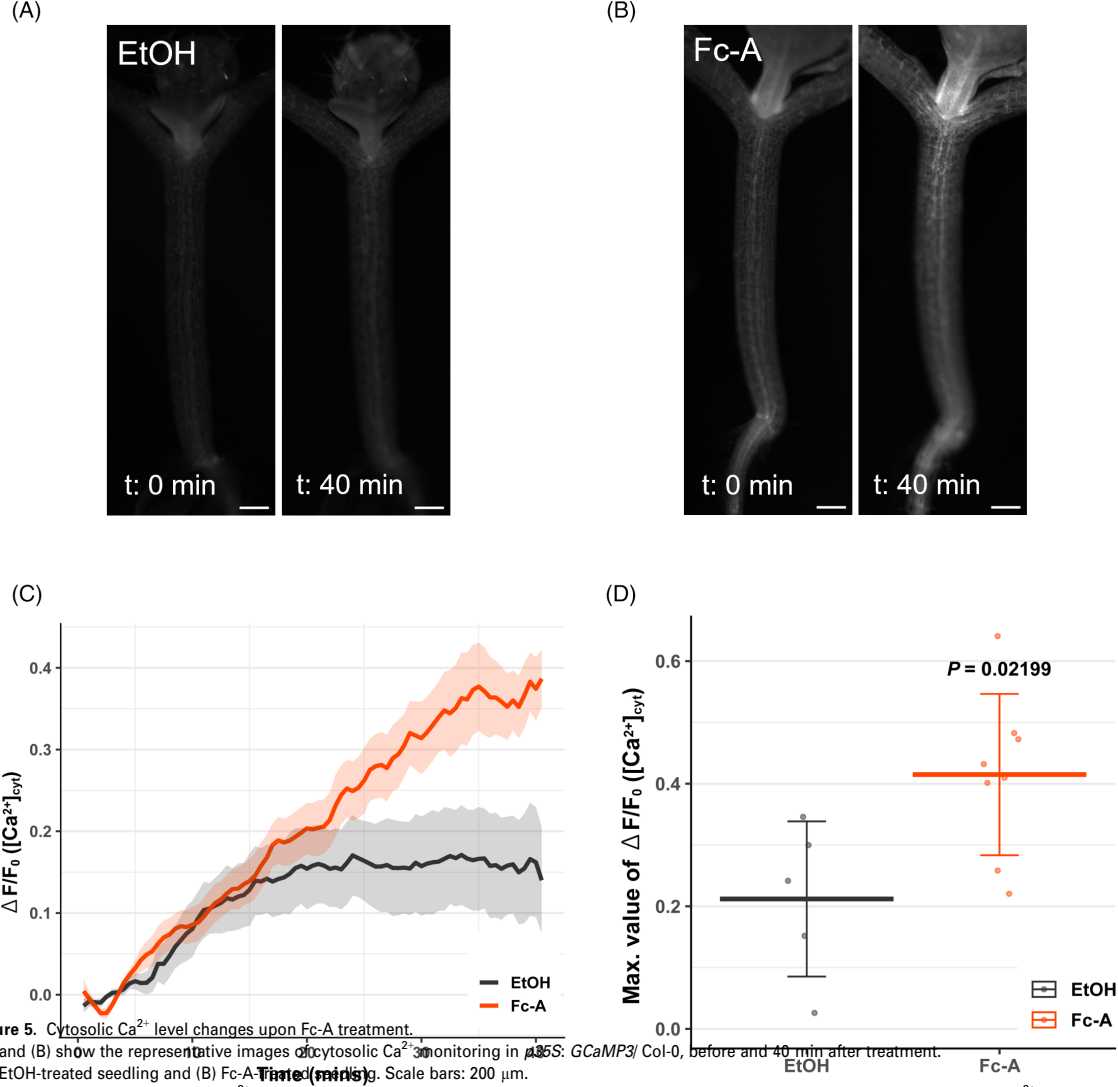
Cytosolic Ca^2+^ level changes upon Fc‐A treatment. (A) and (B) show the representative images of cytosolic Ca^2+^ monitoring in *p35S*: *GCaMP3*/ Col‐0, before and 40 min after treatment. (A) EtOH‐treated seedling and (B) Fc‐A‐treated seedling. Scale bars: 200 μm. (C) The fluorescence of cytosolic Ca^2+^ biosensor was measured at the upper part of the hypocotyl. The measured fluorescence of cytosolic Ca^2+^ biosensor normalised to the fluorescence before the treatment (ΔF/F_0_) along the time after the treatments; the line and the shade represent the mean ± SE in each condition. *n* = 6–7. (D) The comparison of maximum difference (Maximum ΔF/F_0_) in the measurement of (C); Each point represents one biological replicates. Cross‐bars and error bars represent the mean ± SD in each condition. The *P*‐value was determined by Welch's *t*‐test.

**Figure 6:**
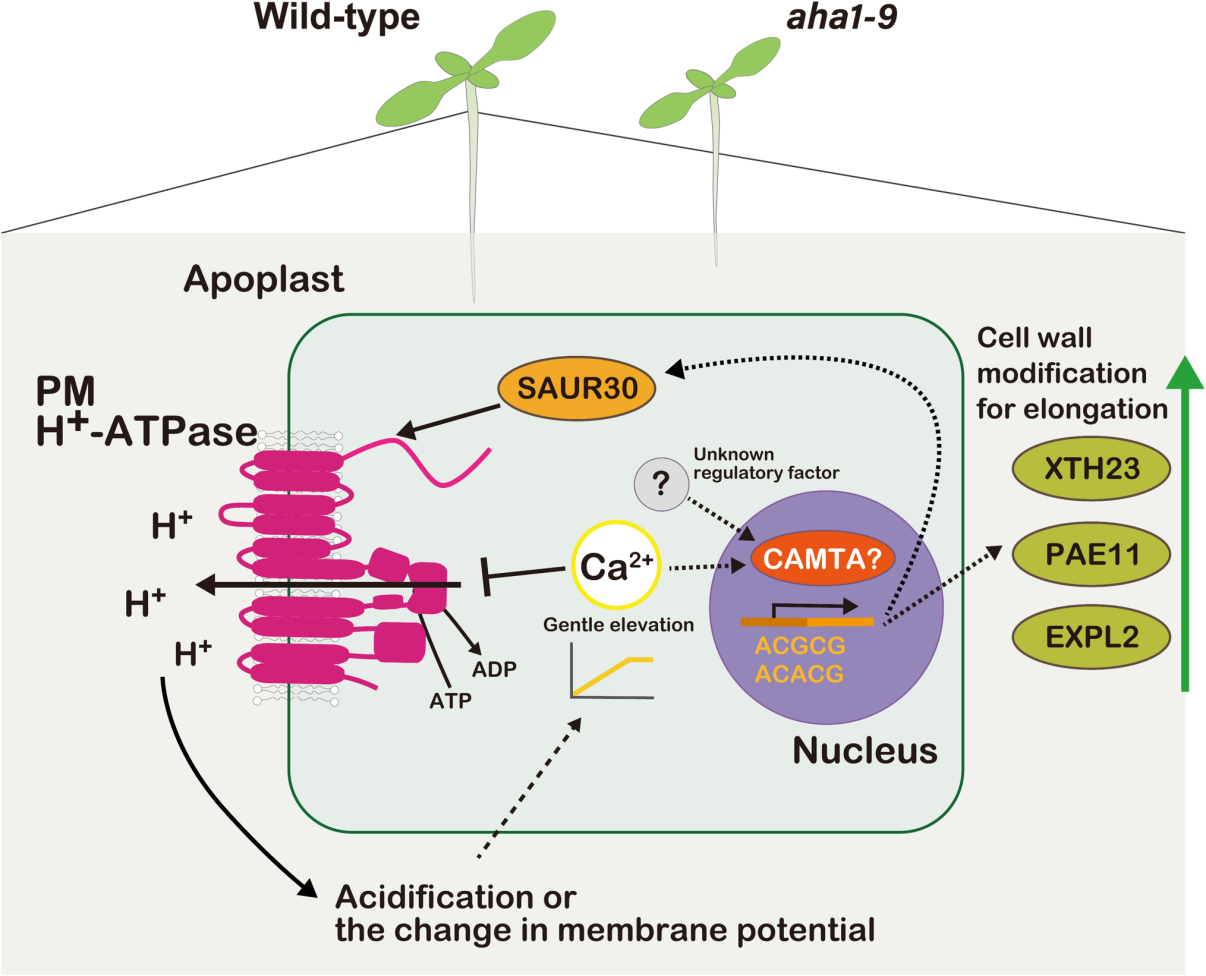
Hypothetical models of PM H^+^‐ATPase activation that triggers cell wall‐related gene expression change in photosynthetic tissues. When PM H^+^‐ATPase is activated, the apoplast becomes acidic and the membrane potential across PM changes. These changes induce the gentle cytosolic Ca^2+^ elevation with an unknown mechanism, and the cytosolic Ca^2+^ elevation and other unknown regulatory factors promote the CAMTA transcription factor. CAMTA induces the expression of cell wall‐related genes, resulting in the modification of cell wall properties that promote the growth of cells. In addition, the elevation of Ca^2+^ reduces the PM H^+^‐ATPase transiently and promotes the expression of an activator of PM H^+^‐ATPase, SAUR30, which may fine‐tune the activity of PM H^+^‐ATPase.

To test whether PM H^+^‐ATPase activation induces the Ca^2+^ elevation in the cytosol as the CAMTA activation requires cytosolic Ca^2+^ elevation, the plants expressing a well‐established Ca^2+^ biosensor, GCaMP3 were treated with Fc‐A or ethanol, and the GCaMP3 fluorescence was monitored. Monitoring the GCaMP3 biosensor revealed that the cytosolic Ca^2+^ levels gradually increased upon Fc‐A treatment compared to the mock ethanol treatments (Figure 5A,B; Video S1). GCaMP3 fluorescence between Fc‐A‐ or ethanol‐treated plants became distinct around 20 min after the treatments (Figure 5C) in rather slow and gentle manner compared to previously known touch‐induced Ca^2+^ spikes (Matsumura et al., 2022), and the maximum changes of GCaMP3 fluorescence were significantly different between Fc‐A‐ and ethanol‐treated plants (Figure 5D), indicating that the PM H^+^‐ATPase activation by Fc‐A invoked the gentle elevation of cytosolic Ca^2+^. These results imply that PM H^+^‐ATPase promotes directly or indirectly the gentle elevation of the cytosolic Ca^2+^ and induces the cell wall‐related gene expression (Figure 6).

## DISCUSSION

### PM H^+^‐ATPase promotes green seedlings' growth by increasing the expression of cell wall modification‐related genes

The idea of acid growth has been accepted for a long time in plant science, while the model is mainly established in non‐photosynthetic tissues, etiolated seedlings and roots. Here, we investigated how PM H^+^‐ATPase activation promotes the growth of green seedling shoots. Our study showed that the growth of photosynthetically active green seedlings is also promoted by PM H^+^‐ATPase, the primary source of ‘acid’ in the apoplast (Figure 1). Our transcriptome analysis revealed that global transcript changes were induced upon PM H^+^‐ATPase activation by Fc‐A treatment (Figure 2). The fungal toxin Fc‐A may induce the transcript changes in other than PM H^+^‐ATPase downstream, as the Fc‐A also activates the potassium channel at the PM, K^+^ *Arabidopsis thaliana* 1 (KAT1) (Saponaro et al., 2017). However, we compared our data with other transcriptome data from the photosynthetic tissue with PM H^+^‐ATPase activating condition to exclude the off‐target effect of Fc‐A. The comparison illustrated that the induction of gene expressions upon PM H^+^‐ATPase activation by Fc‐A on green seedlings, light illumination, or sucrose supplementation on leaves, was enriched in cell wall‐related genes (Figure S5). Among the upregulated cell wall‐related genes, we showed that the induction of *XTH23*, *AGP18*, *EXPL2*, *PAE11*, and *TCH4* expression by Fc‐A depended on PM H^+^‐ATPase (Figure 3). The ectopic overexpression of EXPL2 shows the elongated hypocotyl growth in etiolated seedlings (Boron et al., 2015). XTH23 and TCH4 belong to group 2 of xyloglucan endotransglucosylase/hydrolase family, which modify the flexibility of tissues via remodelling of the xyloglucan tethering structure and may support the cell growth (Rose et al., 2002). For instance, the ectopic overexpression of TCH4 induces the elongation of hypocotyl in etiolated seedlings (Dhar et al., 2022). The deficiency in pectin acetyl esterification by PAE11 leads to reduced photosynthesis performance as well as reduced shoot growth due to the changes in the cell wall components (Roig‐Oliver et al., 2021). In contrast, the ectopic overexpression of arabinogalactan‐protein AGP18 shows the reduced growth of rosette leaves (Zhang et al., 2011), implying that the ectopic overexpression of cell wall‐related genes may cause a problem in cell growth in some cases.

Given that CAMTA proteins regulate the expression of the stress‐regulated transcription factors including the dehydration‐responsive element binding protein 1 (DREB1) family genes and WRKY family genes (Darwish et al., 2022; Kidokoro et al., 2017), it is controversial that CAMTA also plays a role in the cell wall‐related genes to facilitate the growth of cells. Interestingly, a comparison of stress‐related transcription factor gene expression changes in various RNA‐seq datasets from this study and other stress treatment studies revealed that some of the DREB1 family gene expression was not upregulated in the Fc‐A treated samples as strongly as other stress‐treated samples, especially for *DREB1B* gene (Figure S9). The DREB1B is one of the core components in the cold acclimation process as well as other stress acclimation processes, but *DREB1B* upregulation by CAMTA depends on whether the cold stress is rapid or stepwise (Kidokoro et al., 2017). Our dataset comparison result implies that the binding targets of CAMTA may be regulated depending on the states of cells and the amplitude/speed of the signalling input such as cytosolic Ca^2+^ elevation, which may be mediated by association of post‐translational modification or interactor proteins of CAMTA protein. It is also suggested by the recent stomatal guard cell studies that show that the extent and speed of stomatal closure are determined by the number and frequency of Ca^2+^ elevation (Huang et al., 2024).

As a conclusion, although our study has a limitation in proving the effect of cell wall‐related gene expression on the growth of green seedlings, the induction of cell wall‐related genes by PM H^+^‐ATPase activation likely leads to flexible cell wall structure and supports shoot growth (Figure 6). The molecular mechanism of how activation of PM H^+^‐ATPase induces the expression of cell wall‐related genes remains puzzling, but our data suggested the new hypothesis, at least partially, the involvement of Ca^2+^ signalling and CAMTA transcription factor in the upregulation of cell wall‐related genes.

### PM H^+^‐ATPase activation and elevation of cytosolic Ca^2+^

Elevation of cytosolic Ca^2+^ induces the Ca^2+^‐dependent activation of CAMTA transcription factors (Darwish et al., 2022; Finkler et al., 2007). The Fc‐A treatment induced the elevation of cytosolic Ca^2+^ in the hypocotyl of green seedlings (Figure 5). However, how the activation of PM H^+^‐ATPase causes the elevation of cytosolic Ca^2+^ remains elusive. In other words, the connection between pH signalling and Ca^2+^ signalling has been a great target of plant science and discussed for decades (Li et al., 2021a). It is suggested that the increase of cytosolic pH and cytosolic alkalisation invoke the elevation of Ca^2+^ in guard cells (Li et al., 2021a), as well as the apoplast acidification via exposure to low pH buffer also induces the Ca^2+^ signalling in root (Koyama et al., 2001). In contrast, some studies illustrate that the decrease of cytosolic pH upon wounding in leaves induces the elevation of Ca^2+^ (Behera et al., 2018), and a decrease of cytosolic pH via external application of ATP or glutamate induces the spike of Ca^2+^ in the root (Waadt et al., 2020). Our study applied the activator of PM H^+^‐ATPase to green seedlings, managing constitutive pumping of the H^+^ from the cytosol into the apoplast, which leads to an increase of cytosolic pH and a decrease of apoplastic pH concurrently. The pH change across the PM by application of Fc‐A is therefore expected to be physiologically different from other previous studies using the external application of low pH buffer. In line with the hypothesis above, our RNA‐seq analysis also differentiates the amplitude of response between Fc‐A and low pH treatment (Figure 2), suggesting that the activation of the PM H^+^‐ATPase has a more substantial impact on global transcriptome than the application of other external stimuli. In addition, our Ca^2+^ monitoring assay showed the gradual elevation of cytosolic Ca^2+^ and became significantly different at 40 min after the treatment (Figure 5), while other studies with a state‐of‐art engineered genetically encoded biosensor mainly focus on the quick and steep Ca^2+^ elevation within 5–10 min after the treatments (Li et al., 2021a; Waadt et al., 2020). The difference in Ca^2+^ elevation pattern between this study and other studies may come from the tissue specificity and unique application of PM H^+^‐ATPase activator. Thus, this study may provide novel insight into the cytosolic Ca^2+^ signalling mediated by pH changes. However, interpreting the connection between pH change and cytosolic Ca^2+^ spike requires detailed observation, considering the time frame, treatment, and different Ca^2+^ monitoring biosensor systems. Therefore, further investigations are needed to reveal how and what type of Ca^2+^ spike is induced by the PM H^+^‐ATPase activation.

### Complex regulation of PM H^+^‐ATPase activity

Elevation of Ca^2+^ represses the activity of PM H^+^‐ATPase in guard cells (Kinoshita et al., 1995). Some plant Ca^2+^ sensors, SCaBP1 and SCaBP3, participate in repressing PM H^+^‐ATPase activity in seedlings via recruiting the PKS5 kinase (Fuglsang et al., 2007; Yang et al., 2019). Application of rapid alkalisation factor, RALFs, induces the Ca^2+^ spike and repression of PM H^+^‐ATPase, leading to a quick and transient alkalisation of the apoplast in roots, possibly via FERRONIA‐mediated post‐translational regulation of the PM H^+^‐ATPase (Gjetting et al., 2020). The study of RALF also suggests that apoplast acidification induces the expression of RALFs, thus forming negative feedback regulation of PM H^+^‐ATPase. In line with the RALF study, our RNA‐seq confirmed that Fc‐A treatment induced a putative *RALF*, *RALF‐like 33* (AT4G15800; Data S1), as well as identified *SAUR30*, the positive feedback regulators (Figure S7), which has some CAMTA‐binding sites in the promoter (Figure S7B) and phylogenetically belongs to the same clade as Ca^2+^ responsive SAURs (Zhang et al., 2021). Taken together, we propose that upon PM H^+^‐ATPase activation, cytosolic Ca^2+^ elevation induces the quick repression of PM H^+^‐ATPase activity by post‐translational modification and then finely modulates the proper activity of PM H^+^‐ATPase via transcriptional induction of the expression of PM H^+^‐ATPase positive and negative regulators.

### Novel perspective of acid growth model

In the acid growth model, the phytohormones such as auxin and brassinosteroids are the main activators of PM H^+^‐ATPase in the hypocotyl of etiolated seedlings or roots (Miao et al., 2022; Wang et al., 2014), therefore, the acid growth is generally perceived as phytohormone‐dependent phenomena. A recent study illustrates that the activation of PM H^+^‐ATPase in photosynthetic tissues such as leaves is regulated by photosynthetic products (Kinoshita et al., 2023; Okumura et al., 2016). The hypocotyl elongation rate in photosynthetic seedlings is inhibited compared to etiolated seedlings due to the low phytohormone content and reduced sensitivity (Kurepin et al., 2011; Vert et al., 2008). For instance, the ectopic overexpression of PM H^+^‐ATPase activator, small auxin‐up RNAs (SAURs) promote the hypocotyl growth of green seedlings (Kinoshita et al., 2023; Ren & Gray, 2015), suggesting that the PM H^+^‐ATPase activation is a rate‐limitation of the hypocotyl growth in green seedlings. However, the photosynthetic tissue growth, such as leaf expansion, is still maintained at a certain level. Therefore, it is possible that in photosynthetic tissues, the cell growth is supported by photosynthetic product‐dependent activation of PM H^+^‐ATPase as the light illumination as well as sucrose feeding to leaves indeed upregulated essential genes for cell wall modification but not auxin‐ or brassinosteroids‐related genes (Figures S3–S5).

It should be noted that the activation of PM H^+^‐ATPase increases the uptake of nitrate in leaves (Kinoshita et al., 2023). Thus, the impact of PM H^+^‐ATPase activation in green tissues is not limited to the changes in cell wall‐related gene expression but also the facilitation of nutrient translocations in the plant tissues. In addition, the expression of sugar transporter genes and trehalose 6‐phosphate phosphatase genes were uniformly upregulated in Fc‐A‐treated seedlings (Figure S4). The upregulation of the sugar transporters in green tissues may facilitate the sugar storage and translocation from carbon source tissues to promote sink tissue growth (Xu & Liesche, 2021), and upregulation of trehalose‐6‐phosphate phosphatase may increase the content of sucrose under the light condition by fine‐tuning the trehalose‐6‐phosphate‐driven sucrose consumption (Fichtner & Lunn, 2021). Together with the results from this study, we propose adding a new perspective on the acid growth model, shedding light on the impact of PM H^+^‐ATPase activation in photosynthetic tissues, probably mediated by photosynthesis.

## MATERIALS AND METHODS

### Plant materials and growth

The dicot model plant *Arabidopsis thaliana* accession Columbia‐0 (Col‐0) was used as wild‐type, except for the hypocotyl length measurement of *open stomata2‐1D (ost2‐1D)* using Landsberg *erecta* (Ler) as background wild‐type. Seeds were sterilised and stratified with plant preservative mixture (PPM)‐tween solution [2% (v/v) PPM (Plant Cell Technology), 0.005% (v/v) Tween‐20 (Fujifilm)] at 4°C for two nights. After washing, the seeds were sown on 0.8% agar Murashige and Skoog (MS) plate [1/2 MS salt, 0.8% (w/v) Agar (Fujifilm), 2.3 mM MES‐KOH pH 5.7 (Nacalai)] or 0.6% Gellan gum MS [1/2 MS salt, 0.6% (w/v) Gellan Gum (Fujifilm), 2.3 mM MES‐KOH pH 5.7 (Nacalai)] for RNA‐seq samples or other experiments, respectively. The plants were grown under a long day cycle, 16 h light: 8 h dark (6:00 h to 22:00 h light at Japan standard time, JST) at 22–23°C with a photon flux 40–70 μmol m^−2^ s^−1^ of white light. The plates were put horizontally on the shelf to prevent the seedling hypocotyl from touching the plate. The 7‐day‐old seedlings were collected for hypocotyl measurement or transferred to darkness before the treatment of Fusicoccin or EtOH.

### Hypocotyl length and shoot area measurements

For hypocotyl length measurement, the 7‐day‐old seedlings of Col‐0, *aha1‐9* (SAIL_1285_D12; Yamauchi et al., 2016), complementation line *gAHA1*/ *aha1‐9* (Hayashi et al., 2024), Ler, and *ost2‐1D*/ Ler (Merlot et al., 2002, 2007) were transferred and lined onto the agar plate, followed by photograph. The hypocotyl lengths of each genotype in images were manually measured by Fiji software (ImageJ v.1.54). For total green area measurement, the vertically grown 7‐day‐old seedlings of Col‐0, *aha1‐9*, complementation line *gAHA1*/ *aha1‐9*, Ler, and *ost2‐1D*/ Ler were photographed. The shoot area of each seedling was measured using the ‘Colour threshold’ and ‘Analyse Particles.’ functions in Fiji to extract the area of green colour in the picture, which consists of mainly cotyledon and newly emerging leaves with a small part of the hypocotyl.

### Extraction and purification of Fusicoccin‐A

Fusicoccin‐A (Fc‐A) was extracted from *Phomopsis amygdali Niigata* 2 (Sassa et al., 1999), following the procedure in (Sassa et al., 2002). Products were extracted with ethyl acetate, and the crude materials were separated by silica gel column chromatography. Further purification by recrystallisation from ethyl acetate gave FC‐A as a colourless powder, purity >95% on NMR (Kiriyama et al., 2024).

### Treatment on seedling shoot

Plates with 7‐day‐old seedlings were put in a box and kept in the dark overnight before the treatments to reduce the PM H^+^‐ATPase activity. As one biological replicate, 20 seedling shoots were separated from the roots and incubated in the 12‐well plate filled with 1/2 MS liquid media [1/2 MS salt, 2.3 mM MES‐KOH, pH 5.7 except for low pH treatment] containing treatments at final concentration of 0.1% (v/v) ethanol (EtOH), 30 μM Fc‐A in 0.1% ethanol, or liquid media with 2 mM MES‐KOH pH 4.0 (low pH treatment) for 2 h in the dark. The samples were then flash frozen in liquid nitrogen and kept in −80°C until the RNA extractions.

### RNA extraction and cDNA synthesis

Frozen samples were homogenised by beads, and total RNA was purified using the NucleoSpin RNA Plant (MACHEREY‐NAGEL) kit, following the manufacturer's instructions. For real‐time‐quantitative PCR, complementary DNAs (cDNA) were synthesised from 400 ng of purified total RNAs using the PrimeScriptRT Reagent Kit (TaKaRa).

### Preparation of complementary DNA libraries and RNA‐seq analysis

For the preparation of cDNA libraries, the quality of total RNAs was analysed by the Agilent 4150 TapeStation System (Agilent) and then mRNAs were enriched from 600 ng of total RNAs, using Next poly(A) mRNA Magnetic Isolation Module (New England Biolabs, NEB), followed by first‐strand cDNA synthesis with the Next Ultra II RNA Library Prep Kit for Illumina (NEB) and Next Multiplex Oligo for Illumina (NEB). cDNA libraries were then sequenced as single‐end reads for 81 bp on NextSeq 550 system (Illumina). For each treatment condition, three independent biological replicates were sequenced individually and 4.2–6.3 million total reads were obtained. The sequence reads were then mapped to the *Arabidopsis thaliana* reference genome, TAIR10 (Ensemble), with the function ‘RNA‐seq Alignment’ in the web platform, Illumina Basespace (Illumina). Normalisation and statistical analysis for differentially expressed genes were conducted in the web platform, Degust v3.2.0 (Powell, 2019). The differentially expressed genes were defined as log_2_(|foldchange|) >1 and false discovery rate (FDR) = <0.05, compared to EtOH control treatment. For the visualisation of functionally grouped gene expression, MapMan categorical groups (Thimm et al., 2004) were imported from ‘Ath_AGI_LOCUS_TAIR10_Aug2012.txt’ on the website.

### Real time‐quantitative PCR

Primers for amplifying the target genes and internal standard genes were listed in Table S1. Primers and cDNA were mixed with SYBR Green PCR Master Mix (Applied Biosystems) and the fluorescent signals along the cycles were quantified in StepOnePlus™ Real‐time PCR System (Applied Biosystems). The data obtained from the systems was further analysed in Real‐time PCR Miner web platform (Zhao & Fernald, 2005) to calculate the corrected Ct values. The Ct values of target genes were normalised by the Ct value of the internal standard gene, *UBQ5*, to calculate the relative expression of each gene.

### *In silico* promoter analysis

The statistical analysis for overrepresented pentamers in the promoter of the top 200 most Fc‐A‐ and low pH‐induced genes was conducted by following the pentamer prediction programme developed by (Yamamoto et al., 2011). The top 4 most overrepresented pentamers with upstream and downstream were extracted from the datasets (top 200 Fc‐A‐ and low pH‐induced genes) to visualise the frequency of the octamer sequence on the WebLogo web platform (https://weblogo.berkeley.edu/logo.cgi).

### Cytosolic Ca^2+^ imaging

The plate with 7‐day‐old seedlings of Ca^2+^ biosensor line, p35S:GCaMP3 plant (Matsumura et al., 2022; Toyota et al., 2018), were put in a box and kept in the dark overnight. In a dark room, seedlings were transferred to the stage of motorised fluorescence stereomicroscope (SMZ‐25; Nikon) with a 1× objective lens (NA = 0.156, P2‐SHR PLAN APO; Nikon). Live imaging of GCaMP3 fluorescence was conducted using the sCMOS camera (ORCA‐Fusion BT; Hamamatsu Photonics) attached to the stereomicroscope with the NIS‐Elements software (Nikon). After adjusting the focus on the seedling hypocotyl, liquid ½ MS media with 0.1% EtOH or 30 μM Fc‐A was gently applied to the neighbouring region of the seedling, avoiding mechanical contact with the seedling. The fluorescence intensities were quantified using Fiji software (ImageJ v.1.54). The fractional fluorescence changes (Δ*F*/*F*_0_) were calculated using Δ*F*/*F*_0_ = (*F* − *F*_0_)/*F*_0_, where *F*_0_ is the average baseline fluorescence determined by the average of fluorescence intensities in the first 5 min.

### Data analysis, statistics, and graph generation

Most data transformation, statistical analysis, and graph generation were performed in Rstudio (R version 4.3.1) with the packages Tidyverse, multicomp, and ggplot2, except for the MapMan category grouping. The *P*‐value for the MapMan categories was calculated using the Whitney U test in the MapMan software (version 3.0.0).

### Accession numbers

AHA1, AT2G18960; XTH23, AT4G25810; AGP18, AT4G37450; EXPL2, AT4G38400; PAE11, AT5G45280; TCH4, AT5G57560; SAUR30, AT5G53590.

## Author Contributions

SNK and TK initiated and conceptualised the project. SNK and KT conducted most of the experiments except for the cDNA library preparation and RNA‐seq. FO set up the calcium imaging system. JO purified the Fc‐A. MN and YT conducted RNA‐seq and *in silico* promoter analysis. SNK and YH analysed and mapping the raw data from RNA‐seq. YH and KT generated *gAHA1*/*aha1‐9*. SNK prepared the manuscript and figures. IF provided supervision and discussion. All authors reviewed the manuscript.

## Conflict of Interests

The authors declare no competing interests.

## Supporting information


**Dataset S1.** Differentially expressed genes in Fc‐A‐ or low pH‐treated seedling shoot.

**Figure S1.** PM H^+^‐ATPase abundance in the *aha1‐9* mutant and complementation line, *gAHA1*/ *aha1‐9*.
**Figure S2.** Foldchange of transcripts in specified MapMan groups.
**Figure S3.** Comparison of transcript level changes in various stress conditions.
**Figure S4.** Comparison of transcript level changes in various stress conditions with specified MapMan categories.
**Figure S5.** Comparison with photosynthate‐dependent transcriptome change in leaves.
**Figure S6.** Comparison with low pH treatment‐dependent transcriptome change in roots.
**Figure S7.** *SAUR30* expression change in wild‐type and *aha1‐9*.
**Figure S8.** *CAMTA‐*dependent expression profile of cell wall‐related genes.
**Figure S9.** The expression patterns of stress‐induced transcription factors.
**Table S1.** List of primers used in this study.

**Video/Movie S1.** GCaMP fluorescence time laps upon Fc‐A or EtOH treatment.

## Data Availability

The data that support the findings of this study are openly available in DDBJ Sequence Read Archive (DRA) at https://www.ddbj.nig.ac.jp/dra/index‐e.html, reference number BioProject: PRJDB18676.
