# Supplementary material for: Plasma membrane H+‐ATPase activation increases global transcript levels and promotes the shoot growth of light‐grown Arabidopsis seedlings
Source: Plant J. 2025 Feb 7;121(3):e70034. doi: 10.1111/tpj.70034 (PMC11804978; doi:10.1111/tpj.70034)
Supplement: Supplementary file 2 — Figure S1. PM H+‐ATPase abundance in the aha1‐9 mutant and complementation line, gAHA1/ aha1‐9. Figure S2. Foldchange of transcripts in specified MapMan groups. Figure S3. Comparison of transcript level changes in various stress conditions. Figure S4. Comparison of transcript level changes in various stress conditions with specified MapMan categories. Figure S5. Comparison with photosynthate‐dependent transcriptome change in leaves. Figure S6. Comparison with low pH treatment‐dependent transcriptome change in roots. Figure S7. SAUR30 expression change in wild‐type and aha1‐9. Figure S8. CAMTA‐dependent expression profile of cell wall‐related genes. Figure S9. The expression patterns of stress‐induced transcription factors. Table S1. List of primers used in this study. [file TPJ-121-0-s001.pdf]

Supporting Information

Article title: **Plasma membrane H^+-ATPase activation increases global transcript levels and promotes the shoot growth of light-grown Arabidopsis seedlings.**

Authors: Satoru Naganawa Kinoshita, Kyomi Taki, Fumika Okamoto, Mika Nomoto, Koji Takahashi, Yuki Hayashi, Junko Ohkanda, Yasuomi Tada, Iris Finkemeier, Toshinori Kinoshita

The following Supporting Information is available for this article:

Supporting Material and methods

Fig. S1. PM H^+-ATPase abundance in the *aha1-9* mutant and complementation line, *gAHA1*/*aha1-9*.

Fig. S2. Foldchange of transcripts in specified MapMan groups.

Fig. S3. Comparison of transcript level changes in various stress conditions.

Fig. S4. Comparison of transcript level changes in various stress condition with specified MapMan categories.

Fig. S5. Comparison with photosynthate-dependent transcriptome change in leaves.

Fig. S6. Comparison with low-pH treatment-dependent transcriptome change in roots.

Fig. S7. *SAUR30* expression change in wildtype and *aha1-9.*

Fig. S8. *CAMTA*-dependent expression profile of cell wall-related genes.

Fig. S9. The expression patterns of stress-induced transcription factors.

Table S1. List of primers used in this study.

Dataset S1. Differentially expressed genes in Fc-A- or low pH-treated seedling shoot.

Video/Movie S1 GCaMP fluorescence time laps upon Fc-A or EtOH treatment.

Material and methods

SDS-PAGE and Western blot

Plates with 7-day-old seedlings, Col-0, *aha1-9* (SAIL_1285_D12), and complementation line *gAHA1*/ *aha1-9*, were put in box and kept dark for overnight. Shoots of dark treated seedlings were collected in liquid nitrogen and stored in –80°C until SDS-PAGE. Frozen shoots were homogenised with pestle and solubilised in DTT solubilisation buffer [2% (w/v) SDS, 1 mM EDTA, 20% (v/v) glycerol, 10 mM Tris-HCl pH 6.8, 0.012% (w/v) CBB, 50 mM DTT, 1 mM PMSF, 2.5 mM NaF, 20 µM leupeptine]. Identical amounts of lysate supernatant were then loaded to 9% SDS-polyacrylamide gel. After SDS-PAGE, the gel was transferred to nitrocellulose membrane. Total loaded protein content was visualised by PonceauS staining and PM H^+-ATPase protein abundance was detected by custom made anti-PM H+-ATPase (rabbit, 1/3000, Hayashi et al 2012), followed by secondary anti-rabbit-HRP (1/3000).

GO term enrichment

The 235 commonly upregulated genes (fold change > 2) in Fc-A-treated seedlings, light illuminated leaves, and sucrose-fed leaves (Kinoshita et al. 2023) were submitted for GO term enrichment analysis in the web platform, Metascape (Zhou et al. 2019), using the terms from GO Biological Processes.

List of stress-induced transcription factors

The list of 59 stress-induced transcription factors was obtained from UniProt database (https://www.uniprot.org/), using the criteria: "((keyword:KW-0346) AND (keyword:KW-0805) AND (organism_id:3702) AND (go:0003700)) AND (reviewed:true) AND (go:0006950)". KW-0346, Stress response; KW-0805, Transcription regulation; organism_id:3702, Arabidopsis thaliana; go:0003700, DNA-binding transcription factor activity; go:0006950, response to stress.

Fig. S1 PM H^+-ATPase abundance in the *aha1-9* mutant and complementation line, *gAHA1/ aha1-9*.

A representative image of the Western blot analysis, confirming the complementation of PM H^+-ATPase in *gAHA1/ aha1-9*. PM H^+-ATPase abundance was detected by the PM H^+-ATPase specific antibody and total loaded protein amount was visualised by the PonceauS staining of membrane.

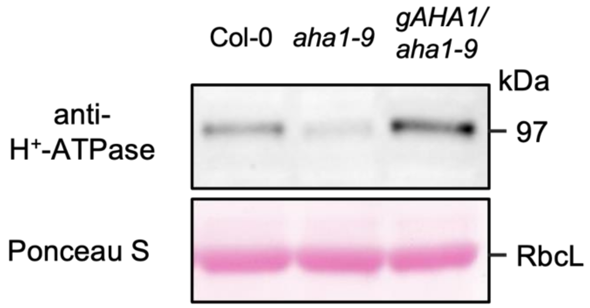

Fig. S2 Foldchange of transcripts in specified MapMan groups

The boxplots represent the distribution of DEGs expression changes in functional category groups compared to the ethanol treated samples. The numbers beside the boxplots indicate the number of DEGs over the number of all registered genes in the category groups. Adjusted *P* values were determined by Mann-Whitney U test in MapMan.

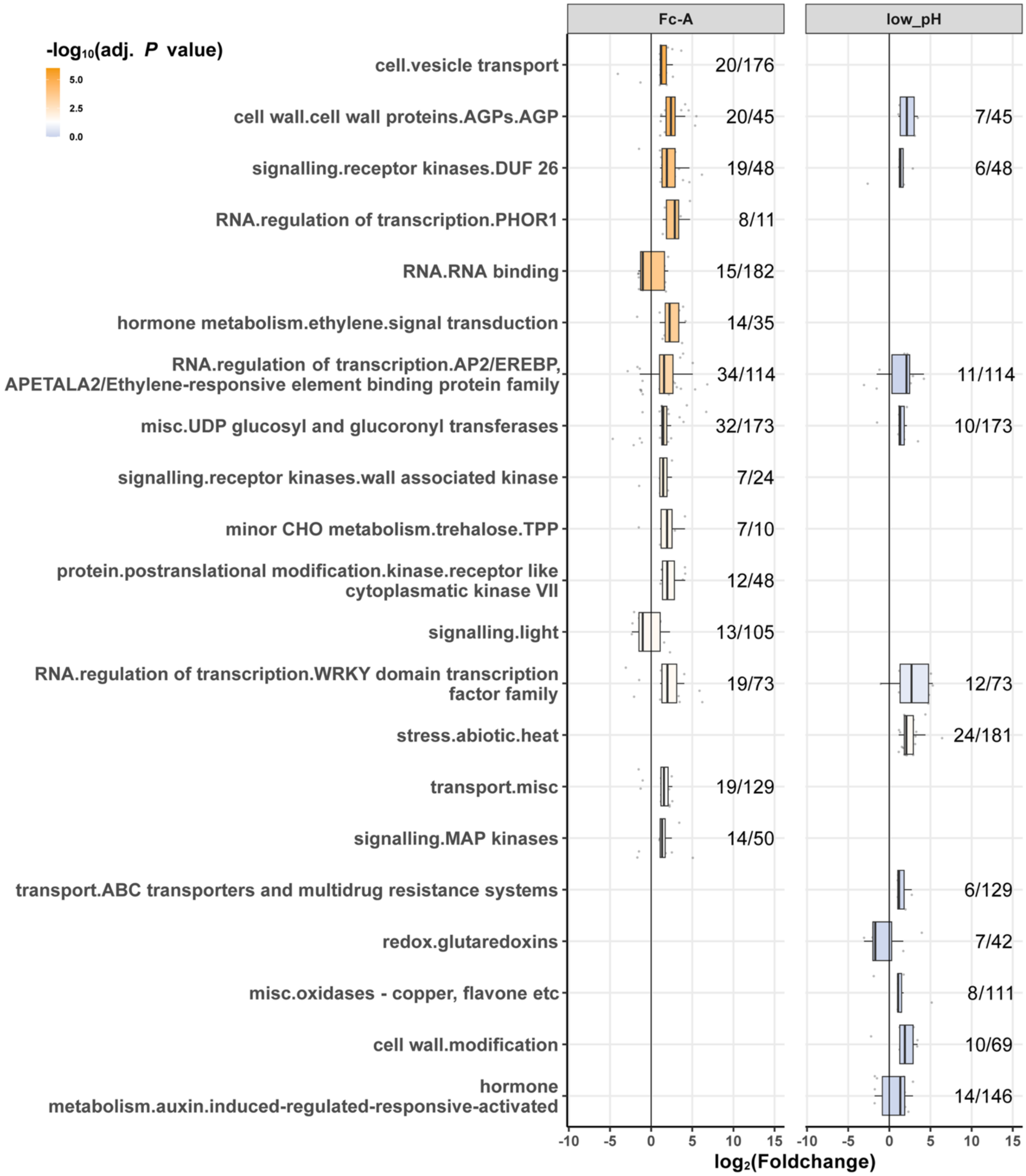

Fig. S3 Comparison of transcript level changes in various stress conditions.

The boxplots represent the distribution of DEGs expression changes in functional category groups compared to the mock samples. The numbers beside the boxplots indicate the percent of DEGs among all registered genes in each category group. The purple gradient colour represents the percent of DEGs in the categories among the total DEGs of each dataset. Col_touch (Ref1) presents the changes in touch-induced DEGs in Darwish et al. 2022. Cold_3hr (Ref2) presents the changes in cold-induced DEGs in Díng et al. 2022. flg22_treated (Ref3) presents the changes in flg22-induced DEGs in Safaeizadeh et al. 2024.

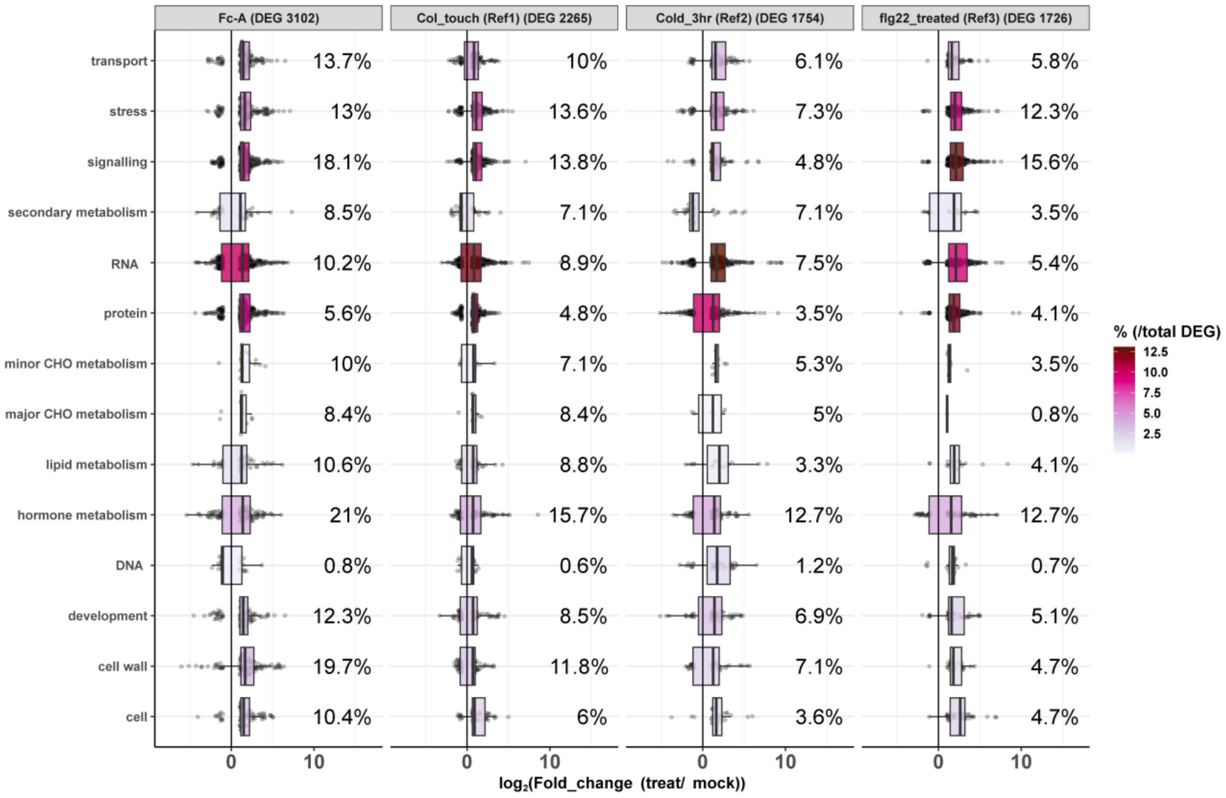

Fig. S4 Comparison of transcript level changes in various stress conditions with specified MapMan categories.

The boxplots represent the distribution of DEGs expression changes in specific functional category groups compared to the mock samples. The numbers beside the boxplots indicate the numbers of DEGs over the numbers of all registered genes in each category group. The purple gradient colour represents the percent of DEGs in the categories among the total DEGs of each dataset. Col_touch (Ref1) presents the changes in touch-induced DEGs in Darwish et al. 2022. Cold_3hr (Ref2) presents the changes in cold-induced DEGs in Díng et al. 2022. flg22_treated (Ref3) presents the changes in flg22-induced DEGs in Safaeizadeh et al. 2024.

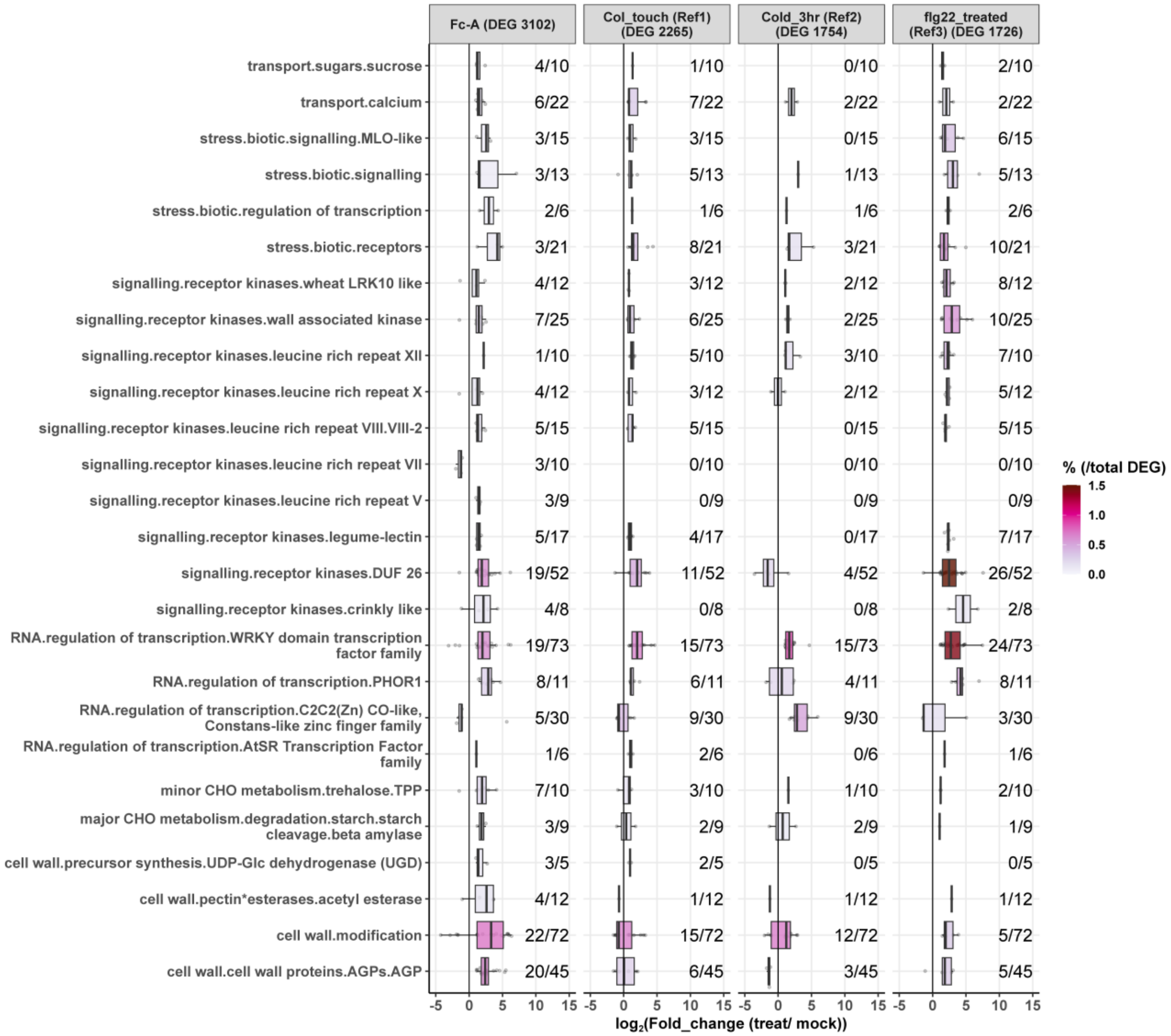

Fig. S5 Comparison with photosynthate-dependent transcriptome change in leaves.

A) Venn diagrams represent the numbers of upregulated transcripts (left; foldchange > 2) or downregulated transcripts (right; foldchange < -2) in Fc-A treated seedlings compared to ethanol treated seedlings, light illuminated leaves or sucrose supplemented leaves from Kinoshita et al. 2023. B) GO term enrichments of commonly upregulated transcripts (235 genes in the panel A).

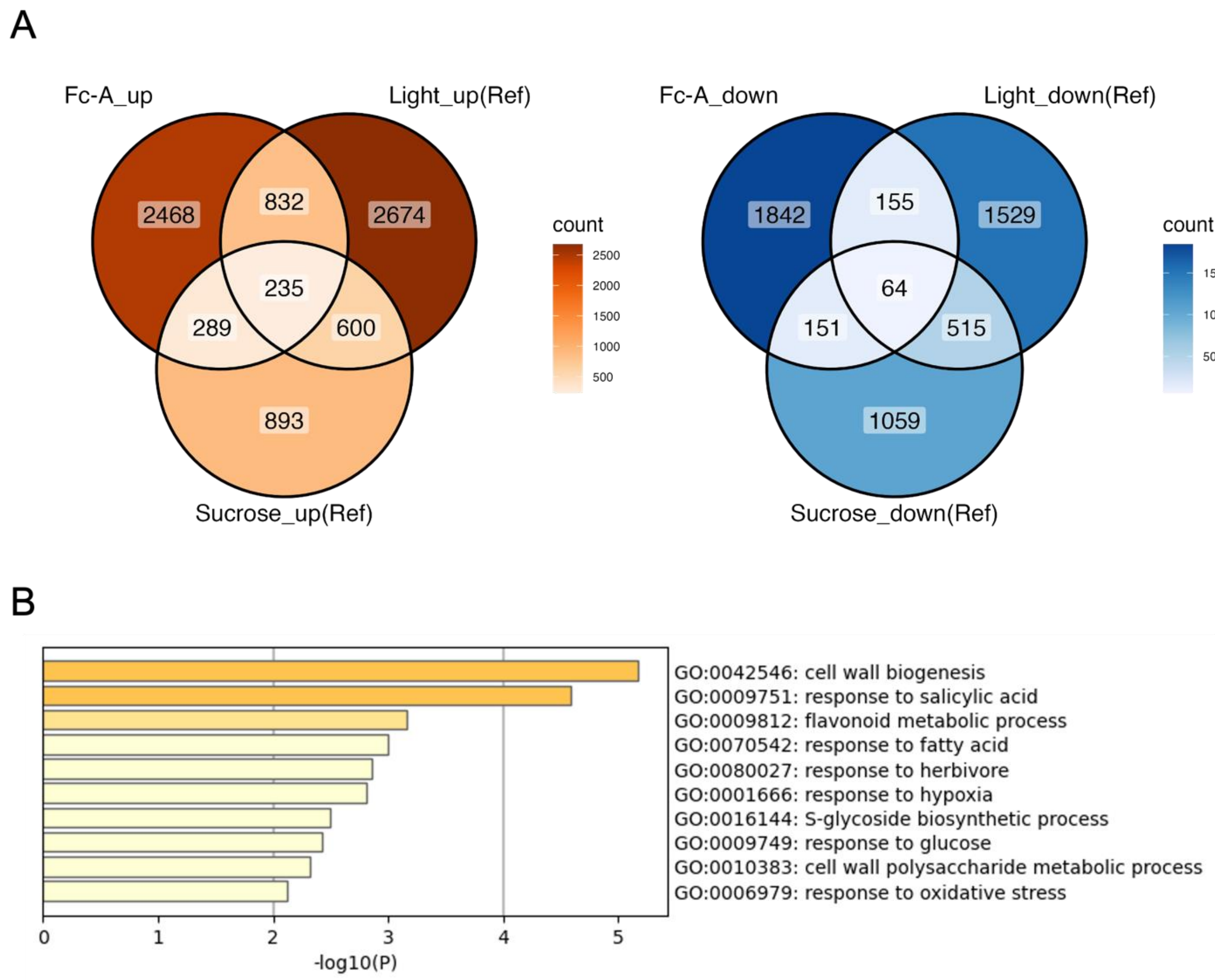

Fig. S6 Comparison with low-pH treatment-dependent transcriptome change in roots.

A) Venn diagram represent the numbers of differentially expressed genes (DEGs; |foldchange| > 2, FDR < 0.05) in Fc-A treated or low pH media treated seedlings compared to ethanol treated seedlings and Ref. data obtained from microarray results from Lager et al. 2010 using low-pH treated roots of Arabidopsis. On the left are upregulated DEGs and on the right are downregulated DEGs. B) Heatmap presents the foldchange (FC) of cell wall-related genes in this study and Ref. data from Lager et al. 2010. Negative control (NC) is EtOH treated seedling shoots. Grey colour in heatmap represent either not significant or not detected in the datasets.

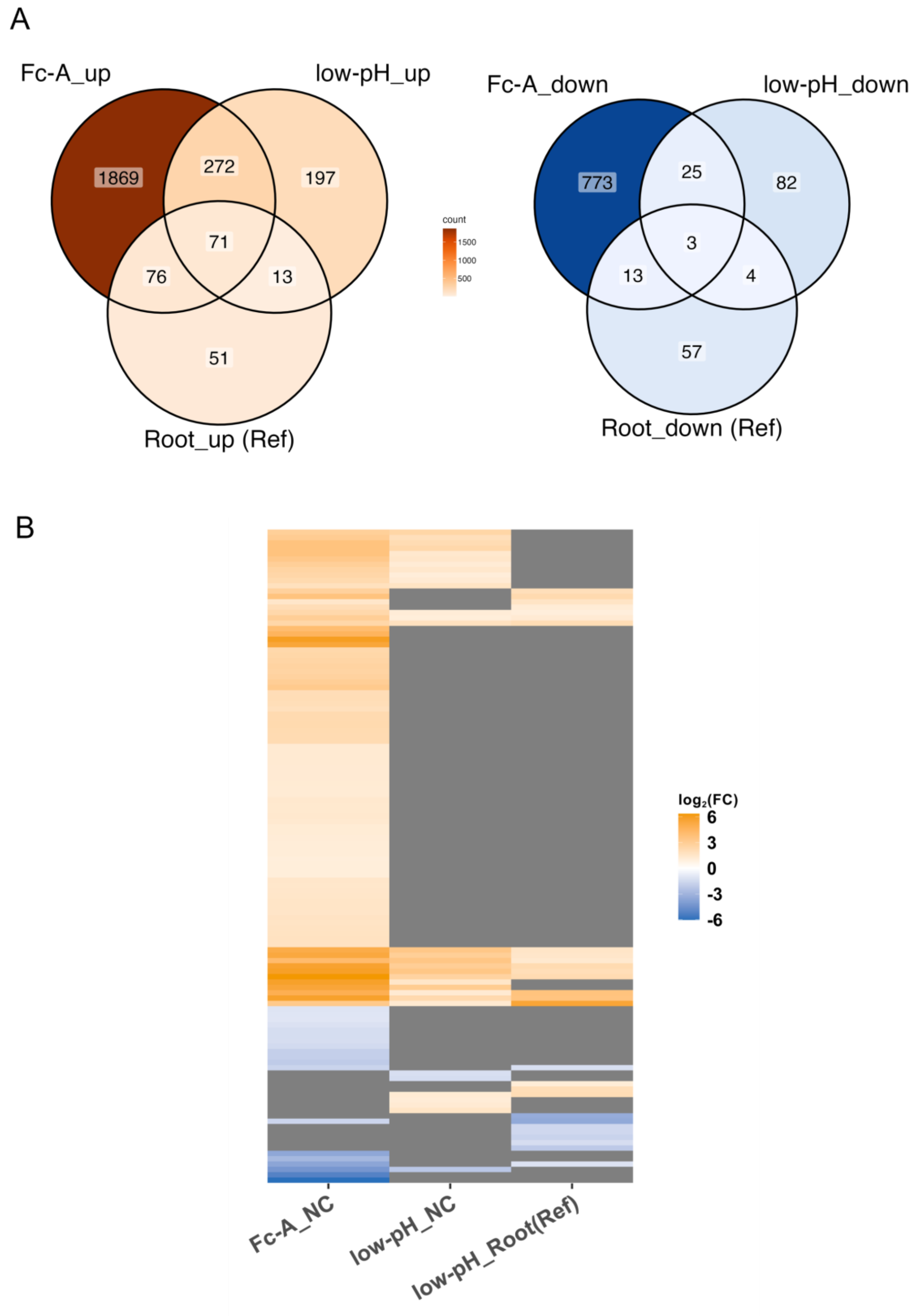

A
Fc-A_up
low-pH_up
1869
272
197
71
76
13
51
Root_up (Ref)
count
1500
1000
500
Fc-A_down
low-pH_down
773
25
82
3
13
4
57
Root_down (Ref)
count
600
400
200
B
log2(FC)
6
3
0
-3
-6
Fc-A_NC
low-pH_NC
low-pH_Root(Ref)

Fig. S7 ***SAUR30*** **expression change in wild type and** ***aha1-9***

A) The relative expression of *small auxin up RNA 30* (*SAUR30*) to ubiquitous *UBQ5* in wildtype (Col-0) and *aha1-9*, determined by RT-qPCR. Each point represents one biological replicates. Crossbars and error bars represent the mean ± S.D. in each condition. Different letters above error bars indicate the significant difference in gene expression, determined by one-way ANOVA with Tukey HSD. B) Schematic diagram of the pentamer positions in the promoter region of *SAUR30* genes. Black line represents the 1000 bp upstream of 5'-UTR; blue narrow line and orange narrow line indicate the position of GCGC box, ACGCG and ACACG pentamer, respectively; white box represents the position of 5'-UTR.

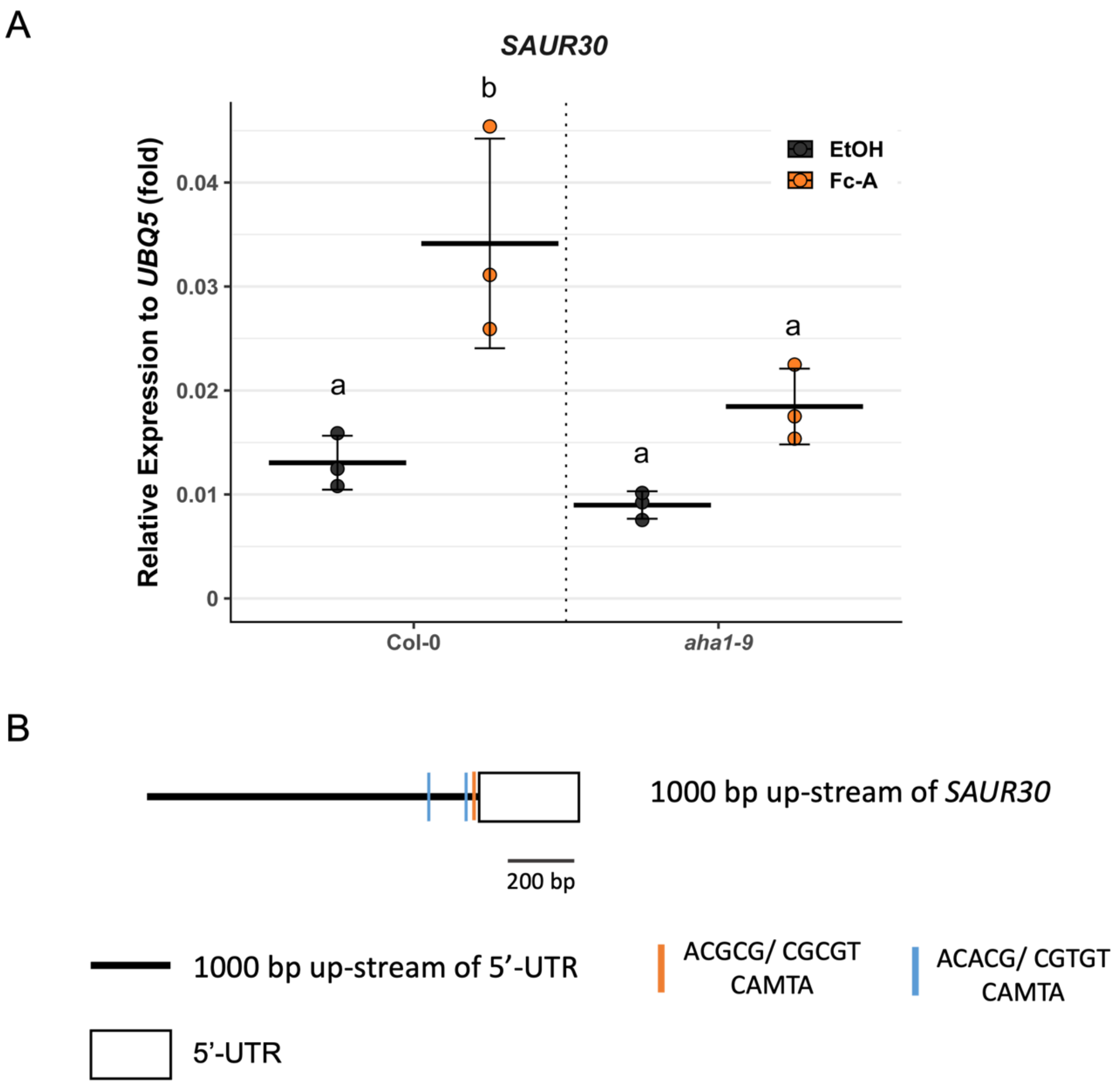

Fig. S8 CAMTA-dependent expression profile of cell wall-related genes

Heatmap presents the foldchange (FC) of cell wall-related genes in this study and Ref. data, *camta* mutant analysis from Kim et al. 2013. Negative control (NC) is EtOH treated seedling shoots. Only detected cell wall-related genes both in this study and Kim et al. 2013 are listed. Blue colour of the AGI number indicates the genes tested in RT-qPCR of this study.

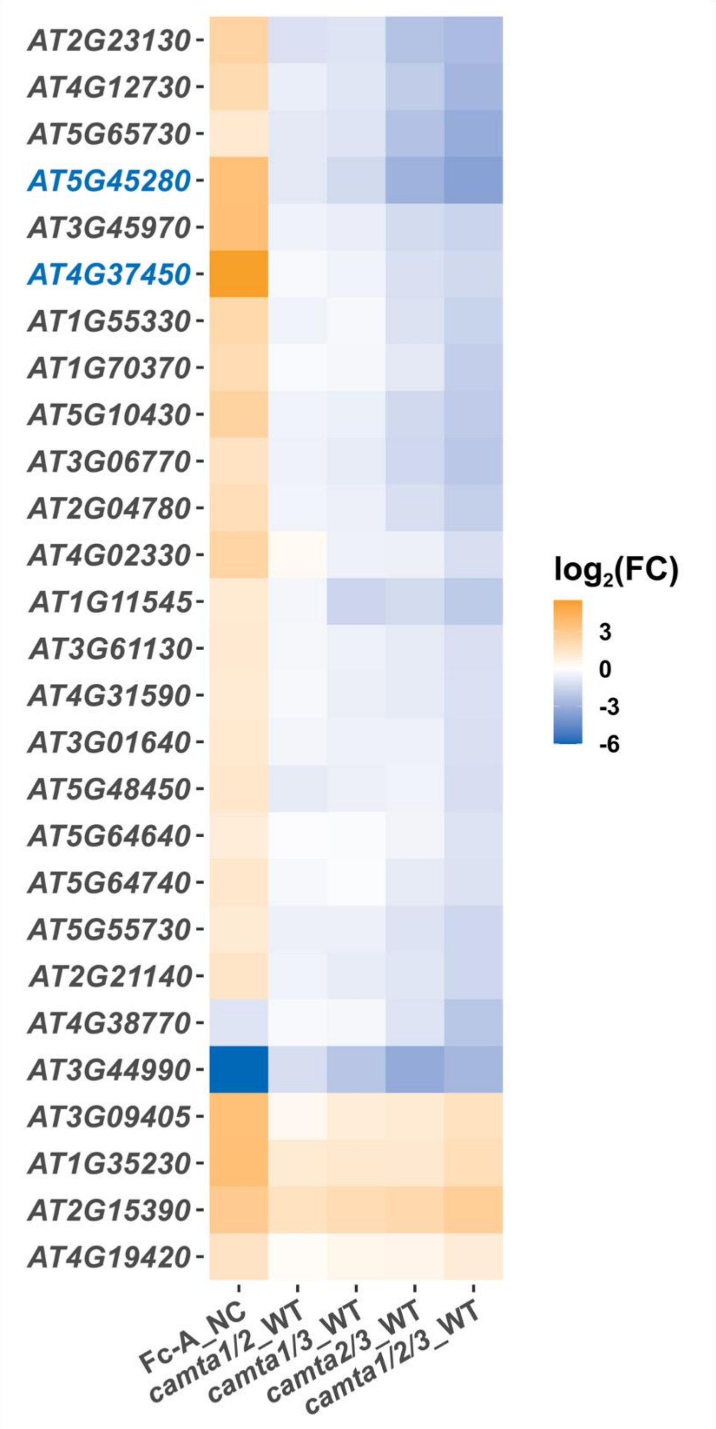

Fig. S9 The expression patterns of stress-induced transcription factors.

Heatmap presents the foldchange (FC) of stress-induced transcription factors (TFs) among the DEGs in this study and reference datasets. The purple gradient colour represents the foldchange of gene expression. The grey colour presents that the gene was not detected as the DEGs in the conditions. Col_touch (Ref1) presents the changes in touch-induced DEGs in Darwish et al. 2022. Cold_3hr (Ref2) presents the changes in cold-induced DEGs in Díng et al. 2022. flg22_treated (Ref3) presents the changes in flg22-induced DEGs in Safaeizadeh et al. 2024.

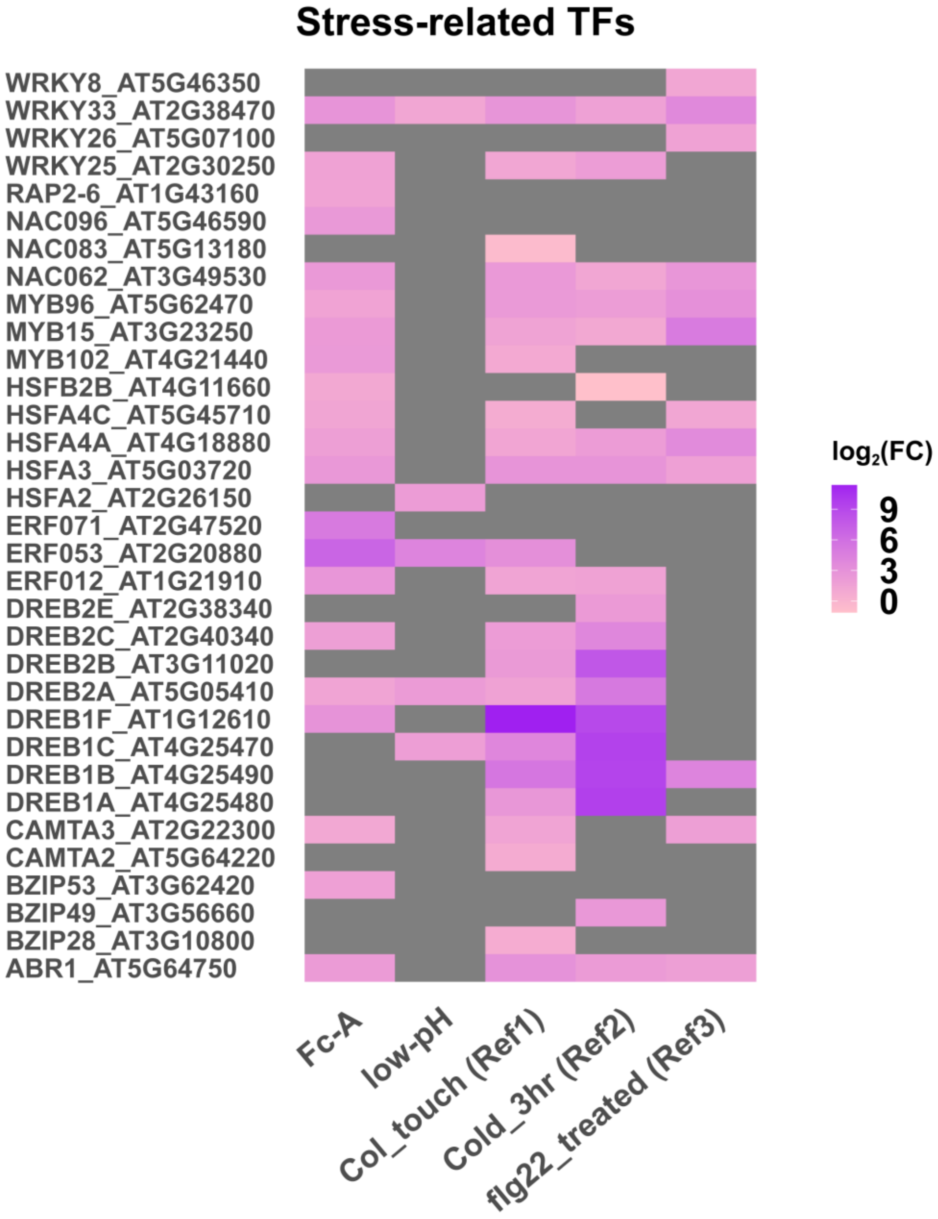

Table S1 List of primers used in this study.

For RT-qPCR		
Name	sequence	target
UBQ5_Fw	CTTGAAGACGGCCGTACCCTC	UBQ5 (internal standard)
UBQ5_Rv	CGCTGAACCTTTCAAGATCCATCG	
XTH23_qP1_Fw	CACCATTGTGGTTGCTCTGC	XTH23
XTH23_qP1_Rv	GATCTGTCCACGTCCGTCTC	
AGP18_qP1_Fw	TGATGTTCTGTTTCTCAACGGC	AGP18
AGP18_qP1_Rv	GCGATGAGGAGAGATCTGGA	
EXPL2_qP2_Fw	TTGTCCTTAGCAGCAGAGCC	EXPL2
EXPL2_qP2_Rv	TTGAGAAGATCTCTGTCGGCG	
PAE11_qP2_Fw	CGCCTTCGATTCTTGGCAGA	PAE11
PAE11_qP2_Rv	GCAGCCAACACTTGGTCTCTAT	
TCH4_qP1_Fw	CACTGCTTCTTACCGTGGCT	TCH4
TCH4_qP1_Rv	CGAGCCAGTAGTAGTCCCCT	
SAUR30_qP6_Fw	TGGTGTTCAAGTTCCACTTCCA	SAUR30
SAUR30_qP6_Rv	CGTGTCCCACCATAATCGCT	

Dataset S1. Differentially expressed genes in Fc-A- or low pH-treated seedling shoots.

Video/Movie S1 GCaMP fluorescence time laps upon Fc-A or EtOH treatment.
Representative live imaging movie of EtOH treated (left) or Fc-A-treated (right) seedling of GCaMP expressing plant. Time stamp represents the time (min : sec) after the application of treatment solution and simultaneous start of imaging. Scale bar, 200 µm.
